# Left Ventricular Diastolic Dysfunction with Elevated Filling Pressures Is Associated with Embolic Stroke of Undetermined Source and Atrial Fibrillation

**DOI:** 10.3390/tomography10100124

**Published:** 2024-10-14

**Authors:** Zubair Bashir, Liqi Shu, Yuqian Guo, Edward W. Chen, Shuyuan Wang, Eric D. Goldstein, Maheen Rana, Narendra Kala, Xing Dai, Daniel Mandel, Shadi Yaghi, Phinnara Has, Mingxing Xie, Tao Wang, James Simmons, Christopher Song, Philip Haines

**Affiliations:** 1 Department of Cardiology, University of Texas Medical Branch, Galveston, TX 77555, USA; 2 Department of Neurology, Alpert Medical School of Brown University, Providence, RI 02903, USA; 3 Department of Anesthesiology and Intensive Care Medicine, The First Affiliated Hospital of Zhejiang University School of Medicine, Hangzhou 310025, China; 4 Department of Internal Medicine, Yale School of Medicine, New Haven, CT 06510, USA; 5 Department of Cardiology, First Affiliated Hospital of Nanjing Medical University, Nanjing 210029, China; 6 Department of Ultrasound, Tongji Medical College, Huazhong University of Science and Technology, Wuhan 430022, China; 7 Department of Neurology, Temple University, Philadelphia, PA 19140, USA; 8 Lifespan Biostatistics, Epidemiology and Research Design, Rhode Island Hospital, Providence, RI 02903, USA; 9 Stanford Cardiovascular Institute, Stanford University, Palo Alto, CA 94304, USA; 10 Department of Pulmonary, Critical Care, and Sleep Medicine, Alpert Medical School of Brown University, Providence, RI 02903, USA; 11 Department of Cardiology, Alpert Medical School of Brown University, Providence, RI 02903, USA

**Keywords:** embolic stroke of undetermined source, atrial fibrillation, left ventricular filling pressure, ischemic stroke

## Abstract

**Background/Objectives**: Left ventricular diastolic dysfunction (LVDD) and elevated left ventricular filling pressure (LVFP) are strong predictors of clinical outcomes across various populations. However, their diagnostic utility in embolic stroke of undetermined source (ESUS) remains unclear. We hypothesized that LVDD with elevated LVFP (based on echocardiography) was more likely to be prevalent in ESUS compared to non-cardioembolic stroke (NCE) and to be associated with atrial fibrillation (AF) on follow-up monitoring. **Methods**: This is a single-center retrospective study that included adult patients with a diagnosis of acute ischemic stroke between January 2016 and June 2017. LV function was assessed by inpatient transthoracic echocardiogram (TTE), and stroke etiology was adjudicated by the neurologist per the consensus criteria. Patients with cardioembolic stroke and those with indeterminate diastolic function on TTE were excluded. Baseline patient characteristics and clinical variables were compared among patients with and without LVDD and elevated LVFP. Multivariable regression models were used to assess the associations between diastolic dysfunction, ESUS, and AF detection in ESUS patients. **Results**: We identified 509 patients with ESUS and NCE stroke who had reported diastolic function. The mean age was 64.19 years, 45.19% were female, and 146 had LVDD with available LVFP data. LVDD was not associated with ESUS (adjusted OR: 1.43, 95% CI: 0.90–2.27, *p* = 0.130) or atrial fibrillation (AF) detection on cardiac monitoring (adjusted OR: 1.88, 95% CI: 0.75–4.72, *p* = 0.179). However, LVDD with elevated LVFP was borderline associated with ESUS (adjusted OR: 2.17, 95% CI: 0.99–4.77, *p* = 0.054) and significantly associated with AF detection (adjusted OR: 3.59, 95% CI: 1.07–12.06, *p* = 0.038). **Conclusions**: Our data suggest that LVDD with elevated LVFP is borderline associated with ESUS and significantly associated with AF detection on follow-up cardiac monitoring. Therefore, the presence of LVDD with an increased probability of elevated LVFP may help identify a subset of stroke patients more likely to have ESUS, potentially due to atrial cardiopathy with underlying occult AF. Further studies are needed to confirm our findings and to evaluate the safety and efficacy of anticoagulation in patients with ESUS and LVDD with elevated LVFP.

## 1. Introduction

Embolic stroke of undetermined source (ESUS) is a non-lacunar ischemic stroke detected on brain imaging without a clear embolic source [1]. It is a subgroup of cryptogenic ischemic stroke, accounting for 17% of all ischemic strokes and impacting approximately 1.3 million people each year [2,3,4]. Potential embolic sources include paroxysmal atrial fibrillation (AF), left ventricular thrombi, ventricular dysfunction, mitral annular calcification, and patent foramen ovale [5]. Despite aspirin monotherapy for secondary stroke prevention, the risk of recurrent stroke after ESUS is approximately 5%, posing a significant clinical dilemma within this patient population [1].

Left ventricular diastolic dysfunction (LVDD) refers to the abnormal relaxation of the left ventricle (LV) during diastole, which may result in impaired LV filling and elevated LV filling pressures (LVFPs) [6]. Previous studies have demonstrated a significant association between LVDD and adverse outcomes following ESUS [7]. However, despite its potential importance, LVDD and elevated LVFP has not been integrated into clinical practice as a risk assessment tool for detecting ESUS-related atrial cardiopathy and underlying occult AF.

We hypothesized that LVDD with elevated LVFP is more likely to be present in patients with ESUS associated with AF on cardiac monitoring compared to non-cardioembolic stroke (NCE). To test this hypothesis, we compared these groups using clinical and echocardiographic data in conjunction with long-term clinical follow-up. We aimed to examine and independently validate a simple LVDD and elevated LVFP-based AF prediction model that may be easily applied in clinical practice.

## 2. Materials and Methods

### 2.1. Data Acquisition

This retrospective study was approved by the Lifespan Institutional Review Board (IRB number: 011317; 45 CFR) with a waiver of informed consent. Data sharing is available on reasonable request.

### 2.2. Patient Population

All included patients were admitted to the Comprehensive Stroke Center of Rhode Island Hospital with a primary diagnosis of acute ischemic stroke between January 2016 and June 2017. Stroke subtypes were classified by a vascular neurologist at discharge using the TOAST classification system [8], and ESUS was diagnosed based on the consensus guidelines as a non-lacunar stroke without evidence of extra- or intracranial arterial stenoses ≥ 50% and without overt major indicators for cardio-embolic stroke or other specific identifiable causes of stroke [5,9]. In addition, patients adjudicated as cardioembolic stroke; those with a left ventricular ejection fraction (LVEF) < 40%, moderate to severe cardiac valve disease, hemodynamically significant pericardial effusion causing tamponade physiology, congenital heart disease, a prosthetic heart valve, or left ventricular assist devices; and those with missing or indeterminate diastolic function data were excluded.

### 2.3. Patient Characteristics

Demographic variables and vascular risk factors, including age and sex, as well as history of hypertension, diabetes mellitus, hyperlipidemia, coronary artery disease, congestive heart failure, renal disease, and active tobacco use, were collected. Laboratory covariates and clinical metrics, such as presenting systolic blood pressure and the National Institutes of Health Stroke Scale (NIHSS) score, were extracted through chart review. Comprehensive ischemic stroke evaluation was performed for all patients, including laboratory tests, neck and head CT angiogram, brain MRI, 12-lead electrocardiogram, and cardiac telemetry monitoring for at least 24 h.

In addition, TTE parameters obtained according to guidelines from the ASE/EACVI were extracted from the clinical echocardiography reports generated by board-certified cardiologists [10]. These parameters included wall motion abnormalities (WMAs); left atrial volume dimensions, including left atrial volume index (LAVI); Doppler-measured tricuspid regurgitation maximum velocity (TR Vmax) and tricuspid peak gradient (TRPG); early and late mitral inflow velocities; and tissue Doppler imaging of mitral annular velocities.

### 2.4. Definition of LVDD and Elevated LVFP

TTE was performed by licensed echocardiography technicians, and quantification of LV diastolic data and filling pressures as well as reporting of LVDD status were performed by board-certified cardiologists according to guidelines from the ASE/ESCVI based on the multiparametric assessment model of determining the status of diastolic function. This included assessing the mitral inflow Doppler (E/A ratio), tissue Doppler imaging (TDI) to measure E′ velocity, the E/E′ ratio, left atrial volume index (LAVI), and tricuspid regurgitation velocity (TR Vmax). The clinical cardiologists used an E/A ratio < 1 or >2 for defining impaired relaxation and restrictive filling, respectively. Moreover, reduced E′ velocities at the lateral and septal annulus (typically < 10 cm/s and <7 cm/s, respectively) were used to comment on impaired myocardial relaxation, and an E/E′ ratio > 14 was reported as suggestive of elevated LVFP [10]. Diastolic dysfunction is graded based on severity. Grade 1 is defined as presence of abnormal myocardial relaxation with normal filling pressures and was determined using E/A < 0.8 with normal or mildly elevated LVFP. As diastolic dysfunction progresses, patients develop a rise in left-sided filling pressures defined as Grade 2 diastolic dysfunction. Determination of Grade 2 diastolic dysfunction with elevated filling pressures is based on using parameters from the multiparametric model from the ASE/ESCVI guidelines and includes the assessment of LAVI, TRVmax, and E/E′ ratios. Some patients progress further to develop restrictive physiology (Grade 3 diastolic dysfunction), which is suggested by echocardiography when E/A > 2, in addition to the presence of the parameters suggesting a higher probability of elevated left-sided filling pressures mentioned above. Progression from Grade 1 to Grade 3 diastolic dysfunction is based on echocardiography criteria and parameters and is associated with a significantly increased risk of cardiac and non-cardiac events, hospitalizations, morbidity, and mortality. We identified subjects with diastolic dysfunction and an increased probability of normal LVFP (Grade 1 diastolic dysfunction) and diastolic dysfunction with an increased probability of elevated LVFP (Grade 2 and Grade 3 diastolic dysfunction) from the echocardiography reports. Of note, echocardiography is able to provide data to determine whether there is an increased probability of elevated LVFP and does not provide a direct measure of LVFP, which would require invasive cardiac catheterization. However, echocardiography is the clinical standard for determining the presence and grade of diastolic dysfunction in the majority of patients.

### 2.5. Outcome

The stroke subtype was determined by inpatient vascular neurologists upon discharge, and ESUS status was defined based on the ESUS consensus criteria [9]. Patients who met the eligibility criteria were monitored for arrhythmia for four weeks with cardiac event monitors on discharge. However, surveillance was extended beyond 4 weeks with an implantable loop recorder (ILR) in a small subset of patients at the discretion of the treating neurologist and cardiologist. The presence of AF was defined as identification of AF for more than 30 s during monitoring [11], and these were confirmed by board-certified cardiologists.

### 2.6. Statistical Analysis

Demographic variables, vascular risk factors, and laboratory characteristics were compared between patients with and without LVDD using the chi-squared test, Fisher’s exact test, the Student’s *t*-test, and the Wilcoxon rank-sum test as appropriate. We also evaluated how these characteristics differed between those without LVDD versus those with LVDD and normal LVFP versus those with LVDD and an increased probability of elevated LVFP.

Using those without LVDD as a reference, multivariable logistic regression analysis was used to assess the association between LVDD and ESUS compared to NCE stroke as well as between LVDD and post-discharge AF detection. To identify potential confounders, backward stepwise logistic regression was performed to identify covariates that significantly differed between patients with and without LVDD.

Furthermore, a three-group comparison was also performed between those without LVDD, those with LVDD and normal LVFP, and those with LVDD and elevated LVFP. Using those without LVDD as a reference, multivariable logistic regression analysis was used to assess the associations between LVDD (with either normal or elevated LVFP) and ESUS compared to NCE stroke. Similarly, we assessed the associations between LVDD (with either normal or elevated LVFP) and post-discharge AF detection. Models were adjusted for variables identified in the aforementioned backward stepwise logistic regression analysis. Additionally, we adjusted for prevalence of congestive heart failure due to its association with elevated LVFP [12].

Additionally, we performed a sensitivity analysis comparing the baseline and echocardiographic characteristics between the two stroke subtypes. An adjusted analysis was also conducted, controlling for covariates that were significantly different between the groups, as identified by stepwise backward logistic regression. These covariates included sex, systolic blood pressure (SBP), left atrial dilation, and LVEF. We did not adjust for the ratio of early-to-late ventricular filling velocities (E/A) or left atrial volume index, as these parameters may be indicative of diastolic dysfunction in a population without underlying AF.

All analyses were performed in STATA (version 18, Stata Corp, College Station, TX, USA), with a *p* value < 0.05 considered statistically significant.

## 3. Results

### 3.1. General Characteristics

**Figure 1:**
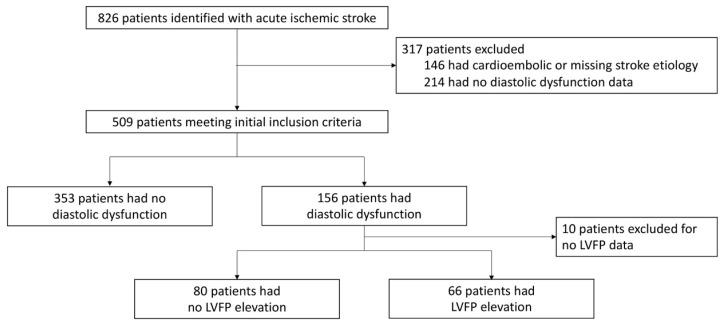
Flowchart of the inclusion and exclusion criteria.

Among 826 patients adjudicated as ESUS and NCE stroke subtypes, 509 patients met the inclusion criteria with reported diastolic function data on clinical echocardiography reports. The mean age was 64.19 years, with 45.19% being females (Figure 1 and Table 1). Among the 509 patients, 146 had LVDD and LVFP data reported, of whom 80 were found to have LVDD and normal LVFP (Grade 1 diastolic dysfunction) and 66 were found to have LVDD and elevated LVFP (Grade 2 or 3 diastolic dysfunction). Clinical characteristics that differed significantly between patients without and with LVDD were age (60.88 ± 14.17 versus 71.69 ± 12.31, *p* < 0.001), chronic arterial hypertension (58.8% versus 81.9%, *p* < 0.001), diabetes mellitus II (21.3% versus 32.9%, *p* = 0.005), hyperlipidemia (36.8% versus 56.8%, *p* < 0.001), coronary artery disease (10.5% versus 23.9%, *p* < 0.001), congestive heart failure (without ejection fraction < 30%) (0.6% versus 6.5%, *p* < 0.001), active tobacco use (35.7% versus 24.0%, *p* = 0.034), LA dilation (5.2% versus 30.5%, *p* < 0.001), and presenting systolic blood pressure (146.03 ± 27.04 mmHg versus 154.29 ± 28.69 mmHg, *p* = 0.003) (Table 1). After backward stepwise regression, age, hypertension, and LA dilation remained significantly different between the two groups. Similar results were found in the three-group comparison (Table 2).

### 3.2. ESUS Association with LVDD and LVFP

Among the 509 patients, 229 were identified as ESUS. Patients with LVDD and elevated LVFP had a higher prevalence of ESUS than patients with LVDD and normal LVFP (68.2% versus 42.5%, *p* < 0.001); in contrast, NCE stroke patients showed the opposite trend. In addition, patients without LVDD had the lowest prevalence of ESUS (39.9%, *p* < 0.001) as opposed to NCE stroke, which showed the highest prevalence in the group without LVDD (Table 2). In the unadjusted logistic regression model, LVDD was significantly associated with ESUS as compared to NCE stroke (OR: 1.95, 95% CI: 1.33–2.85, *p* = 0.001); after adjustment for age and prevalence of hypertension and left atrial dilation, significance was lost (adjusted OR [aOR]: 1.43, 95% CI: 0.90–2.27, *p* = 0.130) (Table 3).

In the three-group analysis, patients without LVDD were used as the reference group. The presence of LVDD and elevated LVFP was significantly associated with ESUS as compared to NCE stroke (OR: 3.22, 95% CI: 1.84–5.64, *p* < 0.001), whereas LVDD and normal LVFP was not significantly associated with ESUS (OR: 1.11, 95% CI: 0.68–1.82, *p* = 0.674). After adjustment for age and prevalence of hypertension, left atrial dilation, and congestive heart failure, the association of LVDD patients and elevated LVFP with ESUS was borderline significant (aOR: 2.17, 95% CI: 0.99–4.77, *p* = 0.054), whereas LVDD and normal LVFP was not significantly associated with ESUS (aOR: 1.03, 95% CI: 0.61–1.77, *p* = 0.903) (Table 4).

In the sensitivity analysis, most of the demographic and medical characteristics were similar between the groups, except for a higher prevalence of females (50.9% vs. 38.8%) and a higher prevalence of congestive heart failure (5% vs. 0.6%) in the ESUS group. Additionally, the median NIHSS score was higher in the ESUS group (11 [IQR 4–19] vs. 7 [IQR 3–15]), whereas the NCE stroke group had a higher mean SBP (152.28 ± 29.38 vs. 144.56 ± 26.4).

Among echocardiographic parameters, the ESUS group had a higher prevalence of left atrial dilation (21.1% vs. 9.1%) and wall motion abnormalities (21.3% vs. 15.0%). Furthermore, the ESUS group exhibited a higher median early-to-late ventricular filling velocity ratio (E/A ratio) (0.98 [IQR 0.76–1.27] vs. 0.86 [IQR 0.7–1.08]) compared to the NCE group. A greater percentage of diastolic dysfunction with elevated LVFP was also observed in the ESUS subgroup (19.72% vs. 6.86%) compared to the NCE stroke group.

After adjusting for covariates that significantly differed between the two stroke subtypes, we found that the odds of ESUS were 2.39 times higher in the presence of LVDD and elevated LVFP compared to the absence of diastolic dysfunction, which was not observed in the NCE subgroup (Supplementary Tables S1 and S2).

### 3.3. AF Detection Association with LVDD and LVFP

Among the 509 patients, 137 received cardiac monitoring, out of whom 55 received ILRs. AF was identified in 26 patients, all of whom were adjudicated as ESUS at the time of discharge. Twelve received ILRs, and the remaining patients received 30-day event monitors. Patients with LVDD and elevated LVFP had a higher prevalence of AF detection as compared to patients with LVDD and normal LVFP (23.5% versus 11.6%, *p* = 0.003). Patients without LVDD had the lowest prevalence of AF detection (7.5%) (Table 2). In the logistic regression model, LVDD was significantly associated with AF detection (OR: 2.72, 95% CI: 1.14–6.48, *p* = 0.024). However, after adjustment for age and prevalence of hypertension and left atrial dilation, LVDD was no longer significantly associated with AF detection (aOR: 1.88, 95% CI: 0.75–4.72, *p* = 0.179) (Table 3).

In the three-group analysis, patients without LVDD were used as the reference group. The presence of LVDD and elevated LVFP was significantly associated with AF detection (OR: 5.86, 95% CI: 1.89–18.17, *p* = 0.002), whereas LVDD and normal LVFP was not significantly associated with AF detection (OR: 1.63, 95% CI: 0.52–5.10, *p* = 0.403). After adjustment for age and prevalence of hypertension, left atrial dilation, and congestive heart failure, the association of LVDD and elevated LVFP with AF detection remained significant (aOR: 3.59, 95% CI: 1.07–12.06, *p* = 0.038), and the association of LVDD and normal LVFP with AF detection remained insignificant (aOR: 1.30, 95% CI: 0.40–4.20, *p* =0.658) (Table 4).

## 4. Discussion

This study provides detailed cardiac and natural history data for a population of non-cardioembolic patients with LVDD and LVFP information. Our study demonstrates a higher prevalence of ESUS in those with LVDD and elevated LVFP (more severe grades of diastolic dysfunction) compared to those with normal diastolic function or with LVDD but normal LVFP (Grade 1 diastolic dysfunction). This indicates that more advanced grades of diastolic dysfunction (LVDD with elevated LVFP) are more strongly associated with ESUS than with the NCE stroke subtype. In addition, our study also showed a higher prevalence of AF detection on mobile cardiac monitoring among ESUS patients with LVDD and elevated LVFP compared to those without LVDD or with LVDD but normal LVFP.

ESUS characterized 45% of all NCE stroke patients, which is similar to the results of previously reported hospital-based studies [2,9,13]. Additionally, the prevalence of AF detection among ESUS patients who completed cardiac monitoring in our study was 22.4%, consistent with previously reported rates ranging from 12.4% to 30% [14,15,16,17].

Our study showed that the assessment of LVDD and LVFP may be an important and easily reportable non-invasive biomarker for future AF detection in ESUS patients, and it may also be used to identify a subset of ESUS patients with a higher risk for a secondary event. Several studies have shown that LVDD is a significant independent risk factor for AF and that the severity of LVDD correlates with the risk for developing AF [18,19,20,21,22]. In addition, the presence of LVDD increases as the severity of AF increases [19]. Our study potentiates this hypothesis by showing a higher prevalence of AF detection among ESUS patients with LVDD and elevated LVFP. The is likely explained by the underlying mechanism of LV wall stiffness and impaired relaxation of LV myocardium during diastole leading to elevated left atrial pressure [23]. This induces morphological changes in the left atrial chamber, including dilatation, fibrosis, and disruption of electromechanical function [24,25,26], causing atrial cardiopathy which can eventually act as a substrate for AF [27,28,29,30].

Identifying atrial fibrillation (AF) as an underlying cause of stroke is crucial for effective stroke prevention, as it often shifts management from antiplatelet to anticoagulation therapy [31]. Various strategies, including serial electrocardiography (ECG) and implantable cardiac monitors (ICMs) based on recommendations from multiple guidelines on cardiac rhythm monitoring in ischemic stroke patients, are used to detect occult AF in ESUS or cryptogenic stroke patients [14,32,33,34,35,36,37]. However, without AF detection, anticoagulation has not proven to be superior to aspirin for stroke prevention in ESUS [38,39]. Although multiple studies have attempted to compare anticoagulation with antiplatelet therapy in cryptogenic stroke patients, these cohorts were limited by significant heterogeneity in their populations [39,40]. Additionally, the recent ARCADIA trial did not reveal any difference between anticoagulation and antiplatelet therapy in reducing recurrent events [41].

As a result, research has shifted towards identifying additional biomarkers of atrial cardiopathy to better identify the specific subset of ESUS patients who may benefit from anticoagulation for secondary prevention. These biomarkers include left atrial (LA) dysfunction, demographics, and anthropometrics [42,43,44]. In this study, we report an additional non-invasive, easily measurable biomarker for early detection of atrial cardiopathy. Previous studies have shown a significant association between LVDD, elevated LVFP, and atrial remodeling in paroxysmal AF, with these factors also being independent predictors of stroke [29,45]. Therefore, when combined with other parameters to detect atrial remodeling, LVDD and elevated LVFP may improve the identification of the ESUS subset with atrial cardiopathy who may benefit from anticoagulation therapy.

Therefore, our research contributes to the understanding of ESUS and atrial cardiopathy and will help to refine the algorithm for risk-stratifying ESUS patients who may benefit from anticoagulation or, at the very least, from intensive arrhythmia surveillance strategies.

### Limitations

Our study, exploring the association between ESUS, LVDD with LVFP, and AF, is subject to several limitations. The single-center nature and modest cohort size may limit the generalizability of our findings, underscoring the need for replication in diverse and larger populations. Moreover, relying on TTE assessments by clinical cardiologists for diagnosing and reporting LVDD and elevated LVFP introduces inherent diagnostic limitations. However, cardiac catheterization, the gold standard for confirming the presence of elevated LVFP is invasive, costly, and impractical for routine clinical practice. Non-invasive echocardiography parameters, such as tissue Doppler imaging, left atrial volume assessment, and Doppler evaluation of mitral valve inflow, offer safe, reproducible, and clinically feasible methods for assessing diastolic function in most clinical settings. Therefore, the practicality and validity of TTE as a surrogate for cardiac catheterization underscore its clinical applicability and support its use for diagnosing LVDD in real-world settings. Furthermore, the retrospective design inherently carries biases and unaccounted confounders, such that prospective studies are needed to confirm our observations and allow for detailed analysis. In addition, the prescription of ILRs and 30-day event monitors varied between the groups, as it was at the discretion of the treating clinician, which again represents a real-world scenario. Moreover, we did not report the burden of AF, which has been shown to be associated with stroke risk. However, it does not influence using strategies such as anticoagulation therapy to prevent secondary events in the absence of any significant comorbidity limiting its use. Notwithstanding these limitations, our findings contribute valuable insights into the role of LVDD with LVFP in predicting AF among ESUS patients, highlighting the importance of echocardiographic assessment in stroke management strategies. Finally, a major strength of our study is that we used the echocardiographic assessment of diastolic function in patients without a confirmed diagnosis of AF, which increased the likelihood that the diastolic dysfunction assessment was accurate. The presence of AF undermines our current ability to provide an accurate echocardiographic assessment of diastolic function and left-sided filling pressures.

## 5. Conclusions

Our study suggests that diastolic dysfunction with elevated LVFP is borderline associated with the ESUS stroke subtype and significantly associated with AF detection on cardiac monitoring. Therefore, the presence of advanced grades of diastolic dysfunction (Grades 2 and 3) may help identify a population of stroke patients more likely to have ESUS, particularly in the setting of occult AF. In addition, it may also aid clinicians in identifying a subset of ESUS patients more likely to have a secondary event due to underlying atrial cardiopathy. Further studies are needed to confirm our findings and to test the safety and efficacy of anticoagulation in a subset of ESUS patients with diastolic dysfunction and elevated LVFP in conjunction with other parameters of atrial cardiopathy.

## Figures and Tables

**Table 1:** Baseline characteristics of patients, stratified by presence of LVDD.

Variable	No LVDD (n = 353)	LVDD (n = 156)	*p* Value
Age (mean ± SD)	60.88 ± 14.17	71.69 ± 12.31	<0.001
Sex (% female)	157/353 (44.5%)	73/156 (46.8%)	0.628
Hypertension (%)	207/352 (58.8%)	127/155 (81.9%)	<0.001
Diabetes (%)	75/352 (21.3%)	51/155 (32.9%)	0.005
Hyperlipidemia (%)	129/351 (36.8%)	88/155 (56.8%)	<0.001
Coronary Artery Disease (%)	37/353 (10.5%)	37/155 (23.9%)	<0.001
Congestive Heart Failure (%) *	2/352 (0.6%)	10/155 (6.5%)	<0.001
Renal Disease (%)	15/352 (4.3%)	13/155 (8.4%)	0.061
Active Tobacco Use (%)	99/277 (35.7%)	23/96 (24.0%)	0.034
Left Atrial Dilation (%)	18/344 (5.2%)	39/128 (30.5%)	<0.001
Systolic Blood Pressure	146.03 ± 27.04	154.29 ± 28.69	0.003
NIHSS (median, IQR)	7 (3–16)	9 (4–17)	0.121
ESUS Stroke (%)	141/353 (39.9%)	88/156 (56.4%)	0.001
NCE Stroke (%)	212/353 (60.1%)	68/156 (43.6%)	0.001
Atrial Fibrillation Detection (%)	17/228 (7.5%)	17/103 (16.5%)	0.012
Wall Motion Abnormalities (%)	29/338 (8.6%)	39/139 (28.1%)	<0.001
Tricuspid Regurgitation Velocity Max (median, IQR)	2.38 (2.17–2.57)	2.51 (2.27–2.86)	<0.001
Left Ventricular Ejection Fraction (median, IQR)	65 (60–67)	65 (55–69)	0.042
Left Atrial Volume (median, IQR)	36 (25–44)	38 (29.5–55)	0.287
Left Atrial Volume Index (median, IQR)	17 (13–22.5)	19.5 (15–27)	0.205
Mitral Valve E/A Ratio (median, IQR)	0.97 (0.76–1.2)	0.84 (0.72–1.09)	0.003
Mitral Valve E/E′ Ratio Septal (median, IQR)	10.38 (8.5–12.62)	14.62 (10.78–18.38)	<0.001
Mitral Valve E/E′ Ratio Lateral (median, IQR)	7.9 (6.23–9.75)	11.85 (8.91–15.32)	<0.001
Mitral Valve E/E′ Ratio Mean (median, IQR)	8.95 (7.31–10.92)	12.89 (9.86–16.17)	<0.001

LVDD: left ventricular diastolic dysfunction, NIHSS: National Institute of Health Stroke Scale, ESUS: embolic stroke of undetermined source, NCE: non-cardioembolic stroke. * Congestive heart failure (ejection fraction > 40%).

**Table 2:** Baseline characteristics of patients without LVDD, patients with LVDD and normal LVFP, and patients with LVDD and elevated LVFP.

Variable	No LVDD (n = 353)	LVDD with Normal LVFP (n = 80)	LVDD with Elevated LVFP (n = 66)	*p* Value
Age (mean ± SD)	60.88 ± 14.17	69.81 ± 11.65	74.58 ± 12.45	0.062
Sex (% female)	157/353 (44.5%)	30/80 (37.5%)	39/66 (59.1%)	0.028
Hypertension (%)	207/352 (58.8%)	59/79 (74.7%)	59/66 (89.4%)	<0.001
Diabetes (%)	75/352 (21.3%)	23/79 (29.1%)	23/66 (34.8%)	0.035
Hyperlipidemia (%)	129/351 (36.8%)	39/79 (49.4%)	42/66 (63.6%)	<0.001
Coronary Artery Disease (%)	37/353 (10.5%)	15/79 (19.0%)	19/66 (28.8%)	<0.001
Congestive Heart Failure (%) *	2/352 (0.6%)	3/79 (3.8%)	7/66 (10.6%)	<0.001
Renal Disease (%)	15/352 (4.3%)	5/79 (6.3%)	7/66 (10.6%)	0.105
Active Tobacco Use (%)	99/277 (35.7%)	15/48 (31.3%)	8/44 (18.2%)	0.068
Left Atrial Dilation (%)	18/344 (5.2%)	7/76 (9.2%)	29/45 (64.4%)	<0.001
Systolic Blood Pressure	146.03 ± 27.04	151.34 ± 27.21	158.03 ± 30.26	0.491
NIHSS (median, IQR)	7 (3–16)	7 (4–17)	11 (4.5–17)	0.019
ESUS Stroke (%)	141/353 (39.9%)	34/80 (42.5%)	45/66 (68.2%)	<0.001
NCE Stroke (%)	212/353 (60.1%)	46/80 (57.5%)	21/66 (31.8%)	<0.001
Atrial Fibrillation Detection (%)	17/228 (7.5%)	5/43 (11.6%)	12/51 (23.5%)	0.003
Wall Motion Abnormalities (%)	29/338 (8.6%)	20/75 (26.7%)	16/54 (29.6%)	<0.001
Tricuspid Regurgitation Velocity Max (median, IQR)	2.38 (2.17–2.57)	2.4 (2.21–2.63)	2.77 (2.4–2.96)	<0.001
Left Ventricular Ejection Fraction (median, IQR)	65 (60–67)	65 (55–69)	62 (55–70)	0.149
Left Atrial Volume (median, IQR)	36 (25–44)	34 (20–43)	43 (36–57)	0.018
Left Atrial Volume Index (median, IQR)	17 (13–22.5)	16 (12–21)	21 (19–33)	0.006
Mitral Valve E/A Ratio (median, IQR)	0.97 (0.76–1.2)	0.75 (0.67–0.84)	1.04 (0.85–1.27)	<0.001
Mitral Valve E/E′ Ratio Septal (median, IQR)	10.38 (8.5–12.62)	12 (9.47–14.62)	17.8 (15.4–19.5)	<0.001
Mitral Valve E/E′ Ratio Lateral (median, IQR)	7.9 (6.23–9.75)	9.47 (8–11.9)	14.7 (12.74–17.7)	<0.001
Mitral Valve E/E′ Ratio Mean (median, IQR)	8.95 (7.31–10.92)	10.38 (8.53–12.89)	15.68 (14–18.29)	<0.001

LVDD: left ventricular diastolic dysfunction, NIHSS: National Institute of Health Stroke Scale, ESUS: embolic stroke of undetermined source, NCE: non-cardioembolic stroke, LVFP: left ventricular filling pressure. * Congestive heart failure (ejection fraction > 40%).

**Table 3:** Multivariable logistic regression analysis of LVDD with ESUS outcome and AF detection in a population of ESUS and NCE stroke patients.

	No LVDD	LVDD
**ESUS**		
Unadjusted OR (95% CI)	Reference	1.95 (1.33–2.85) *p* < 0.001
Adjusted ^†^ OR (95% CI)	Reference	1.43 (0.90–2.27) *p* = 0.130
**AF Detection**		
Unadjusted OR (95% CI)	Reference	2.72 (1.14–6.48) *p* = 0.024
Adjusted ^†^ OR (95% CI)	Reference	1.88 (0.75–4.72) *p* = 0.179

LVDD: left ventricular diastolic dysfunction, ESUS: embolic stroke of undetermined source, NCE: non-cardioembolic, AF: atrial fibrillation. ^†^ Adjusted for age and prevalence of hypertension and left atrial dilation.

**Table 4:** Multivariable logistic regression analysis of LVDD (stratified by LVFP) for ESUS outcome and AF detection in a population of ESUS and NCE stroke patients.

	No LVDD	LVDD with Normal LVFP	LVDD with Elevated LVFP
**ESUS**			
Unadjusted OR (95% CI)	Reference	1.11 (0.68–1.82) *p* = 0.674	3.22 (1.84–5.64) *p* < 0.001
Adjusted ^§^ OR (95% CI)	Reference	1.03 (0.61–1.77) *p* = 0.903	2.17 (0.99–4.77) *p* = 0.054
**AF Detection**			
Unadjusted OR (95% CI)	Reference	1.63 (0.52–5.10) *p* = 0.403	5.86 (1.89–18.17) *p* = 0.002
Adjusted ^§^ OR (95% CI)	Reference	1.30 (0.40–4.20) *p* = 0.658	3.95 (1.07–12.06) *p* = 0.038

LVDD: left ventricular diastolic dysfunction, LVFP: left ventricular filling pressure, ESUS: embolic stroke of undetermined source, AF: atrial fibrillation. ^§^ Adjusted for age and prevalence of hypertension, left atrial dilation, and congestive heart failure.

## Data Availability

Data sharing is available on reasonable request to the corresponding author.

## Supplementary Materials

The following supporting information can be downloaded at: https://www.mdpi.com/article/10.3390/tomography10100124/s1, Table S1: Baseline characteristics of patients, stratified by stroke subtype; Table S2: Multivariable logistic regression analysis of LVDD (stratified by LVFP) for ESUS Outcome.
