# Supplementary material for: Left Ventricular Diastolic Dysfunction with Elevated Filling Pressures Is Associated with Embolic Stroke of Undetermined Source and Atrial Fibrillation
Source: Tomography. 2024 Oct 14;10(10):1694–705. doi: 10.3390/tomography10100124 (PMC11511054; doi:10.3390/tomography10100124)
Supplement: Supplementary file 1 [file tomography-10-00124-s001.zip › tomography-3193135-supplementary.pdf]

Table S1. Baseline characteristics of patients, stratified by stroke subtype.

	NCE	ESUS	*p*-Value
Age (mean ± SD)	64.64 ± 13.97	66.23 ± 14.83	0.152
Sex (% female)	137/353 (38.8%)	164/322 (50.9%)	0.002
Hypertension (%)	238/351 (67.8%)	222/322 (68.9%)	0.751
Diabetes Mellitus (%)	87/349 (24.9%)	91/322 (28.3%)	0.329
Hyperlipidemia (%)	153/349 (43.8%)	158/322 (49.1%)	0.175
Coronary Artery Disease (%)	51/351 (14.5%)	47/322 (14.6%)	0.981
Congestive Heart Failure (%)	2/350 (0.6%)	16/322 (5.0%)	<0.001
Renal Disease (%)	18/349 (5.2%)	22/322 (6.8%)	0.360
Active Tobacco Use (%)	94/276 (34.1%)	70/253 (27.7%)	0.112
Left Atrial Dilation (%)	31/340 (9.1%)	60/284 (21.1%)	<0.001
Systolic Blood Pressure (%)	152.28 ± 29.38	144.56 ± 26.4	<0.001
NIHSS Score (median, IQR)	7 (3–15)	11 (4–19)	<0.001
Atrial Fibrillation detection (%)	2/137 (1.5%)	39/322 (12.1%)	<0.001
Wall Motion Abnormality (%)	50/333 (15.0%)	63/296 (21.3%)	0.041
Tricuspid Regurgitation Velocity Max (median, IQR)	2.43 (2.2–2.65)	2.47 (2.24–2.74)	0.123
Early ventricular filling velocity (median, IQR)	0.78 (0.65–0.94)	0.78 (0.66–0.98)	0.658
Late ventricular filling velocity (median, IQR)	0.89 (0.74–1.07)	0.8 (0.63–1)	<0.001
Early diastolic filling deceleration time (median, IQR)	216 (192–252)	207 (175.5–242)	0.301
Left Ventricular Ejection Fraction (median, IQR)	65 (60–70)	65 (60–65)	0.015
Left Atrial Volume (median, IQR)	36 (25.5–43)	37 (28–52)	0.302
Left Atrial Volume Index (median, IQR)	17 (14–21)	20 (14–26)	0.234
Mitral Valve E/A Ratio (median, IQR)	0.86 (0.7–1.08)	0.98 (0.76–1.27)	<0.001
LA Reservoir Strain (median, IQR)	32.8 (24.78–43.59)	28.21 (19.58–37.76)	<0.001
LA Contractile Strain (median, IQR)	15 (10–22)	13 (9–20)	0.006
LA Conduit Strain (median, IQR)	17 (11–24)	15 (9–21)	0.001
LVDD status			<0.001
No LVDD (n)	212/277	141/218	
LVDD without elevated LVFP (n)	46/277	34/218	
LVDD with elevated LVFP (n)	19/277	43/218	

NCE: Non cardioembolic, ESUS: Embolic stroke of undetermined source, NIHSS: National Institute of Health Stroke Scale, LVDD: Left ventricular diastolic dysfunction, LVFP: Left ventricular filling pressure

Table S2. Multivariable Logistic Regression Analysis of LVDD (Stratified by LVFP) for ESUS Outcome.

	No LVDD	LVDD with normal LVFP	LVDD with elevated LVFP
		ESUS	
Adjusted§ OR (95% CI)	Reference	0.99 (0.57–1.97) *p* = 0.969	2.39 (1.06–5.39) *p* = 0.036

ESUS: embolic stroke of undetermined source, LVDD: left ventricular diastolic dysfunction, LVFP: left ventricular filling pressure. §Adjusted for sex, systolic blood pressure, left atrial dilation, and left ventricular ejection fraction.
